# Effect of Surface Nanocrystallization on Wear Behavior of Steels: A Review

**DOI:** 10.3390/ma17071618

**Published:** 2024-04-01

**Authors:** Khashayar Morshed-Behbahani, Zoheir Farhat, Ali Nasiri

**Affiliations:** Department of Mechanical Engineering, Dalhousie University, 1360 Barrington St., Halifax, NS B3H 4R2, Canada; zoheir.farhat@dal.ca

**Keywords:** wear, steel, stainless steel, surface nanocrystallization, surface severe plastic deformation

## Abstract

Ferrous alloys, particularly steels, form a specialized class of metallic materials extensively employed in industrial sectors to combat deterioration and failures caused by wear. Despite their commendable mechanical properties, steels are not immune to wear-induced degradation. In this context, surface nanocrystallization (SNC) technologies have carved a distinct niche for themselves by enabling the nanostructuring of the surface layer (with grain sizes < 100 nm). This process enhances overall mechanical properties to a level desirable for wear resistance while preserving the chemical composition. Existing literature has consistently highlighted the efficacy of various SNC methods in improving the wear resistance of ferrous alloys, positioning SNC as a promising tool to extend materials’ service life in practical applications. This review provides a comprehensive examination of the SNC techniques employed in surface treatment of ferrous alloys and their impact on wear behavior. We delved into the underlying mechanisms governing wear in SNC-treated Fe-based alloys and concluded with a discussion on current challenges and future perspectives in this evolving field.

## 1. Introduction

Ferrous alloys are metallic materials primarily based on iron (Fe) and are categorized into two main groups: steels (with a carbon content of less than 2.11 wt.%) and cast iron (with a carbon content exceeding 2.11 wt.%) [1]. These alloys find extensive applications in critical industries such as aerospace, construction, automotive, and chemical processing [2]. Among these, steels are the most widely used engineering alloys due to the abundant presence of iron in the Earth’s crust, their diverse mechanical properties, and the ability to undergo solid-state phase transformations, resulting in microstructural evolution [1].

Steels can be further classified into two major categories: low-alloy (comprising low, medium, and high-carbon steels) and high-alloy steels. High-alloy steels, in turn, are subcategorized into tool steels and stainless steels. Stainless steels, defined by their chromium (Cr) content of at least 12.00 wt.%, are further classified into five distinct families based on their resulting microstructure, including austenite, ferrite, and martensite phases [3].

Wear, the process of material removal from a solid surface due to impact or friction [4], is a significant issue in industries such as mining and metal processing, leading to material degradation. Consequently, it imposes financial burdens on nations through loss of materials, equipment downtime for repairs, and eventual replacement of worn-out parts [5]. Furthermore, wear-induced degradation poses a unique challenge for industries like nuclear, where the release of nuclear waste could lead to severe casualties and substantial economic ramifications [6].

In light of these challenges, steels are extensively employed across various industries due to their ease of manufacture and the favorable mechanical properties achieved via phase transformations [7]. While special grades of steel, such as tool steels [8], are engineered for superior mechanical properties and wear resistance, they still experience wear and degradation under real-world service conditions. For example, reports indicate that wear resulted in the degradation of H13 steel dies during the extrusion of pure nickel [9]. Similarly, fastener clips made from 60Si2Mn steel for high-speed railway tracks have been found to suffer from fretting [10]. Indeed, a plethora of cases [11,12,13,14,15] can be identified in the existing literature where Fe-based materials have failed due to wear.

Wear, primarily that occurring at the interface of rubbing surfaces, prompts the need to modify the mechanical properties of the ferrous alloys’ surfaces for improved wear resistance. Various techniques, including cryotreatment [16], laser cladding [17], and laminar plasma jet surface hardening [18], have been employed for this purpose. Notably, surface nanocrystallization (SNC) technologies, as will be thoroughly discussed in the subsequent section, have the capacity to refine grains within the surface layer of alloys to dimensions as minuscule as a few tens of nanometers. This refinement can lead to improvements in overall mechanical properties while preserving the alloy’s chemical composition [3].

The corrosion behavior of surface nanocrystallized (SNCed) stainless steels has been extensively reviewed and explored in the literature [3]. However, the influence of SNC methods on the wear behavior of ferrous alloys has remained largely unaddressed. Despite the prevalent use of SNC technologies since the early 2000s, their impact on the wear response of Fe-based alloys has not been sufficiently emphasized. Therefore, this review aims to comprehensively explore and assess various SNC approaches employed in surface modification across all ferrous materials documented in the literature, shedding light on how these methods can affect wear resistance. Subsequently, we will summarize the wear behavior and underline the mechanisms governing the wear characteristics of these metallic materials. Finally, we will provide a concise overview of current challenges associated with SNC-treated ferrous alloys and outline potential future research directions in this field.

## 2. Summary of Wear Mechanisms

**Figure 1:**
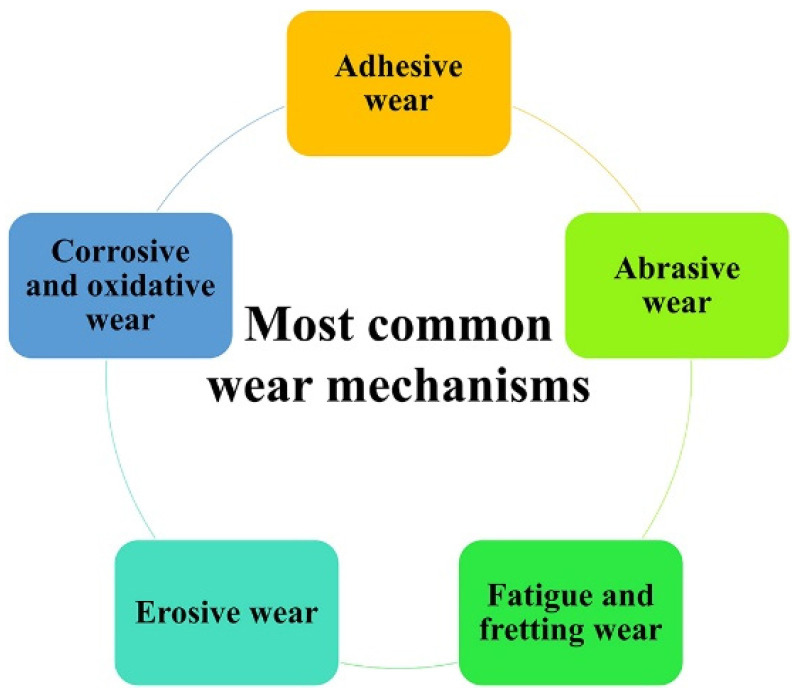
Major wear mechanisms.

Before delving into the wear characteristics of SNC-treated ferrous alloys, it is recommended to review a summary of wear mechanisms depicted in Figure 1. Notably, the five mechanisms presented in Figure 1 represent the most widely recognized wear mechanisms reported in the literature. For a concise reference, Table 1 summarizes these key wear mechanisms that influence the behavior of materials. Nonetheless, for detailed information, readers are encouraged to refer to the cited reference [4], as this review primarily focuses on the impact of SNC rather than an extensive discussion of wear mechanisms.

## 3. Surface Nanocrystallization Technologies

**Figure 2:**
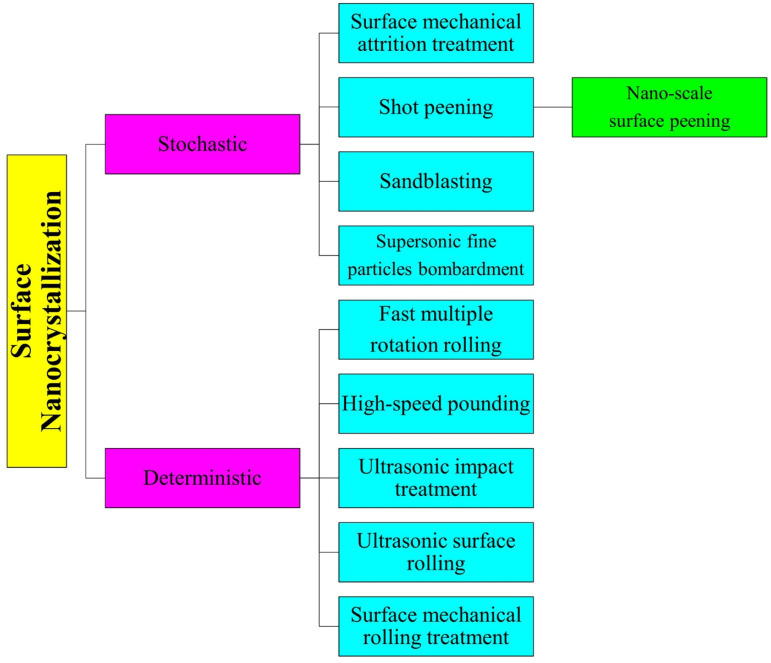
Classification of the methods used to create ferrous alloys with nanostructured surfaces.

The categorization of SNC techniques applied to modify ferrous alloys is presented in Figure 2. It is important to highlight that despite a previous study [3] demonstrating the feasibility of indirect methods capable of surface modification without direct contact between the alloy surface and the tool tip, such as cavitation peening, for the surface treatment of stainless steels, Figure 2 delineates that, thus far, only direct technologies have been utilized to generate nanocrystalline surface layers on ferrous alloys. The direct stochastic approaches involve particles or shots impacting the alloy surface in a non-uniform manner, leading to random surface modifications. On the other hand, direct deterministic methods employ controlled tool tips that deform the surface layer in a precise and regulated manner. Table 2 aims to provide readers with a concise and accurate description of these utilized techniques, offering a technological overview before delving into the wear responses of alloys treated using these methods.

## 4. Summary of Studies on the Influence of SNC on the Wear of Steels

This section comprehensively reviews and discusses the effects of SNC on the wear properties of various ferrous alloys. These alloys have undergone surface modification using a range of SNC technologies. It is salient to note that all the information presented in this section, along with additional details about the material grades and surface roughness, will be consolidated and summarized into a comprehensive table in the following section for easy reference.

### 4.1. Surface Mechanical Attrition Treatment (SMAT)

**Figure 3:**
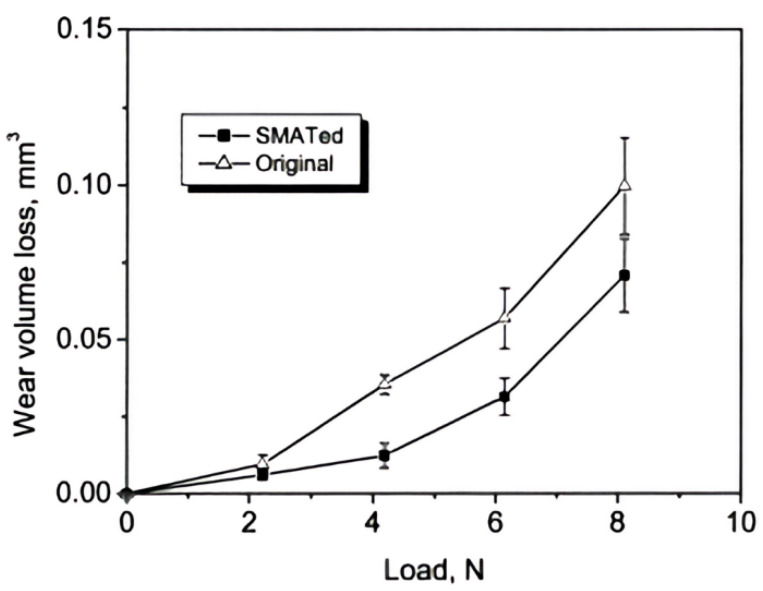
Wear volume loss variations with load for the original low-carbon steel samples and the SMATed specimens [30].

In an earlier study conducted by Wang et al. [30], surface mechanical attrition treatment (SMAT) using hardened steel balls with a diameter of 8 mm was employed to produce a nanostructured surface layer in low-carbon steel. Their findings indicated that the surface layer of the SMAT-treated alloy, characterized by a grain size of approximately 15 nm, resulted in a reduction in friction coefficient and an increase in wear resistance compared to its as-annealed counterpart (see Figure 3). At lower loads, the improvement in friction and wear behavior was attributed to the higher hardness of the nanocrystalline surface layer, which reduced the extent of plowing and micro-cutting. Likewise, at higher loads, the reduced degree of plastic removal and surface fatigue fracture, both stemming from the increased hardness of the surface layer, contributed to the enhanced tribological performance [30].

**Figure 4:**
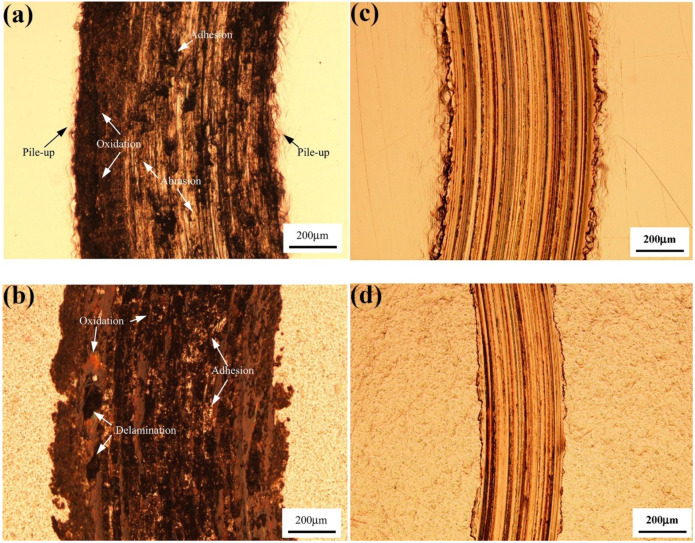
Optical micrographs depicting the wear track on the (**a**,**c**) as-received and (**b**,**d**) SMATed 304 stainless steel alloy samples fabricated under 20 N load: (**a**,**b**) without and (**c**,**d**) with oil lubrication [31].

The wear behavior of SMATed ferrous alloys, using AISI 440 stainless steel balls with a diameter of 6 mm, has been shown to be influenced by sliding conditions. In the study [31], it was observed that under dry conditions, the wear response of SMATed 304 stainless steel alloy did not significantly differ from that of the untreated alloy. Both experienced material removal from the wear track in the form of debris, primarily due to abrasion, adhesion, delamination, and oxidation processes. In this scenario, the untreated sample underwent significant hardening during sliding, resulting in a hardness level similar to that of the SMATed alloy. This similarity in hardness explains their comparable wear characteristics (Figure 4a,b). However, when lubrication was introduced during sliding, the primary mechanism for material removal from the wear track shifted to plastic deformation, mainly driven by the plowing action of the slider. In this context, the SMATed sample, with its higher hardness, exhibited enhanced wear resistance compared to the untreated alloy (Figure 4c,d) [31].

**Figure 5:**
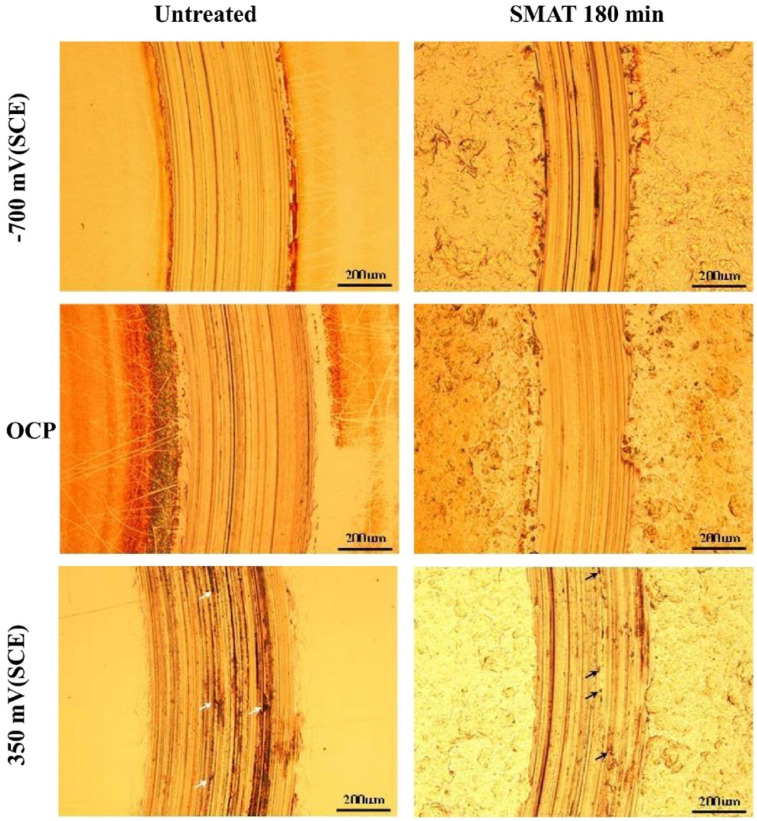
Micrographs depicting the surface morphology of the wear tracks formed on the untreated and SMATed 304 stainless steel samples at the cathodic overpotential of −700 mV (SCE), at open circuit potential (OCP), and the anodic overpotential of +350 mV (SCE). Sliding condition: 0.9% NaCl, 20 N, 60 rpm, 4500 s. Arrows indicate micropits inside the track [32].

In addition to studying wear under dry conditions, research has also delved into the tribocorrosion behavior of SMATed ferrous alloys. For instance, the tribocorrosion resistance of 304 austenitic stainless steel was investigated in a 0.9 wt.% NaCl solution under different electrochemical conditions, including open circuit potential (OCP), cathodic overpotential, and anodic overpotential (Figure 5) [32]. The findings [32] revealed that no corrosion occurred at a cathodic overpotential of −700 mV_SCE_, indicating pure mechanical wear that resulted in surface degradation (Figure 5). Chemical wear initiated at the OCP contributed to approximately 10% of the material removal (Figure 5), and this effect on surface deterioration was intensified significantly when an anodic overpotential of +350 mV_SCE_ was applied (Figure 5) [32].

Notably, the worn tracks observed for SMATed samples, which were surface-modified using stainless steel balls with a diameter of 6 mm, appeared narrower than those of their untreated counterparts (Figure 5) [32]. Consequently, the activated area exposed to the corrosive solution was reduced, leading to smaller electrical currents. This phenomenon can be explained by the hardening effect induced by SMAT, effectively decreasing mechanical wear. In simpler terms, the hardened surface layer developed by SMAT on the alloy reduced mechanical wear, resulting in a smaller area exposed to the NaCl solution and, consequently, reduced tribocorrosion [32].

**Figure 6:**
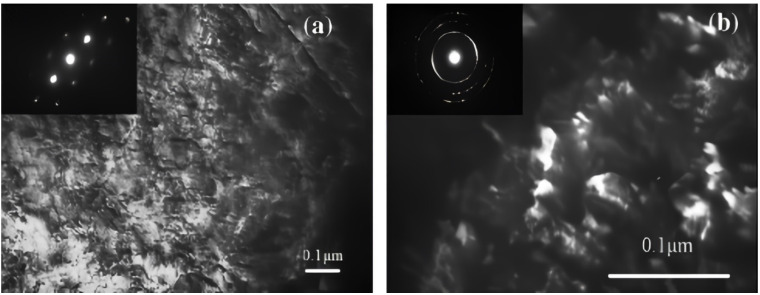
Images taken with a dark field plane-view TEM of the top surfaces of (**a**) un-SMAT and (**b**) SMAT 321 stainless steel samples after plasma nitriding at 4 h for 400 °C [33].

The post-treatment process has also been demonstrated to significantly alter the wear properties of surface nanocrystallized alloys. In a specific case [33], AISI 321 stainless steel underwent SMAT using stainless steel balls of 3 mm diameter and was subsequently subjected to low-temperature nitriding. This treated alloy’s wear resistance was compared to that of an untreated counterpart subjected to plasma nitriding. Microscopic examinations unveiled several noticeable defects in the nitrided untreated specimen (see Figure 6a), while a coarse-grained S-phase structure was detected within the α matrix of the nitrided SMAT-treated sample featuring a nanostructure (see Figure 6b).

**Figure 7:**
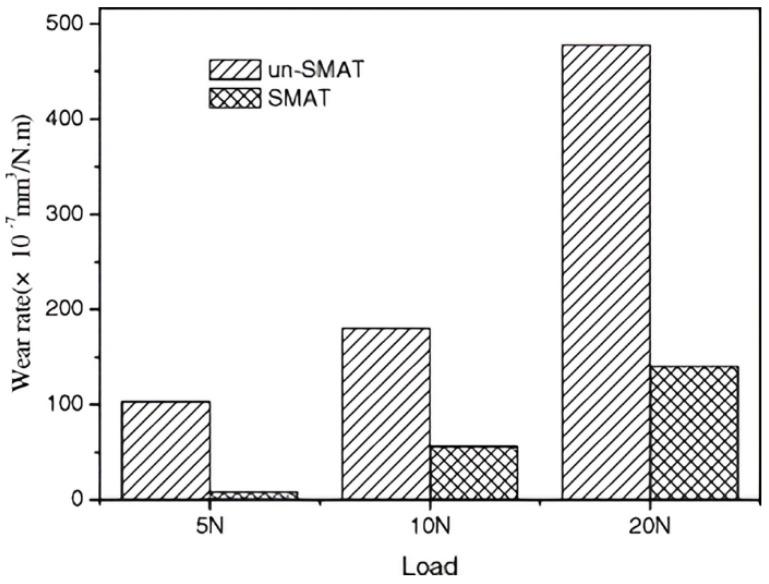
Wear rate for SMAT and un−SMAT 321 stainless steel specimen plasma nitrided at 400 °C for 4 h as a function of the applied force [33].

Consequently, the wear resistance of the SMAT-treated specimen proved to be 3–10 times higher than that of the untreated alloy (refer to Figure 7) [33]. This notable improvement was attributed to the increased hardness and enhanced load-bearing capacity of the SMAT-treated sample, resulting from a thicker nitrided layer and a gradual gradient in the hardness profile of the nitrided layer. Furthermore, it was reported that the fracture failure, such as spalling and chipping, observed in the untreated specimen was a consequence of severe deformation exceeding the tensile strength of the S phase. In contrast, a tribochemical reaction occurring on the surface of the SMAT-treated alloy effectively mitigated the real contact between the slider and the nitrided surface, leading to superior wear resistance [33].

### 4.2. Shot Peening (SP)

**Figure 8:**
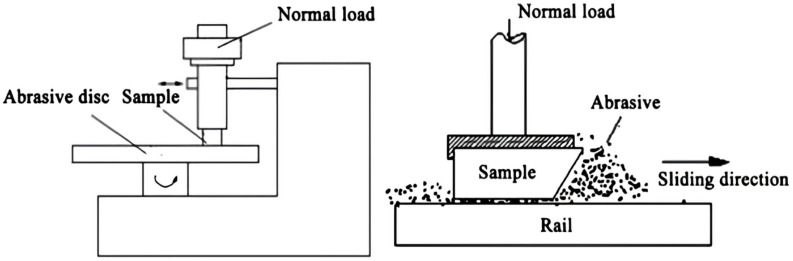
Wear geometry diagram of two-body (emery paper) and three-body (glass sand) abrasive wear [34].

Variations in shot peening duration have been found to be effective in altering the wear behavior of ferrous alloys. Yan et al. [34] reported that the process of surface nanocrystallization, using 0.2 mm diameter cast steel, in Hadfield steel can lead to the formation of a 100 µm thick nanostructured surface layer with grain sizes ranging from 11.1 to 17.4 nm, which can significantly impact wear behavior. In this study, two-body and three-body abrasive wear tests were conducted, as depicted in Figure 8, using emery paper and glass sand, respectively [34].

**Figure 9:**
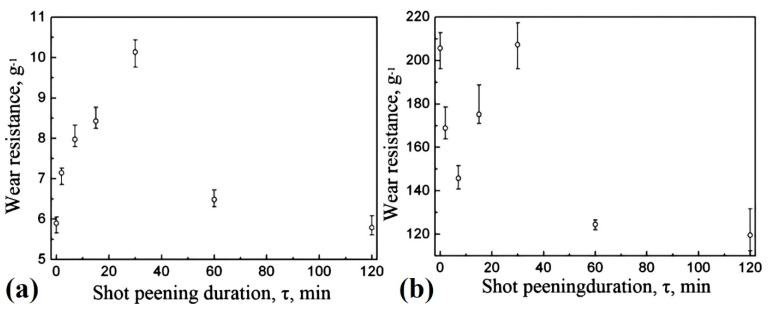
Wear resistance relative to shot peening treatment duration using (**a**) glass particles and (**b**) bonded emery particles as abrasives [34].

For three-body abrasive wear, increasing the shot peening duration to a certain threshold improved wear resistance, as shown in Figure 9a [34]. However, exceeding this threshold deteriorated wear properties. Conversely, two-body abrasive wear showed that shot peening and the subsequent increase in its duration did not contribute to an enhancement in wear resistance, as shown in Figure 9b since the hardness of the emery paper (1800 HV) was greater than that of the shot peened sample. This behavior of glass abrasive three-body abrasive wear was attributed to the increased hardness of the shot-peened steel’s surface, which effectively prevented the penetration of abrasive particles into the non-nanocrystallized subsurface bulk material. Although shot peening increased surface hardness and yield strength and thereby boosted wear resistance, prolonged treatment resulted in microcrack formation, adversely impacting the wear resistance of the steel [34].

In a subsequent study, Yan et al. [35] explored the influence of impact load on the wear resistance of shot-peened Hadfield steel. The findings revealed that a light impact load moderately improved wear resistance, while heavy impact loads did not enhance it. Furthermore, it was suggested [35] that prolonged shot peening had a detrimental effect on wear resistance, primarily due to the formation of micro-cracks. This observation aligns with their earlier findings [34].

**Figure 10:**
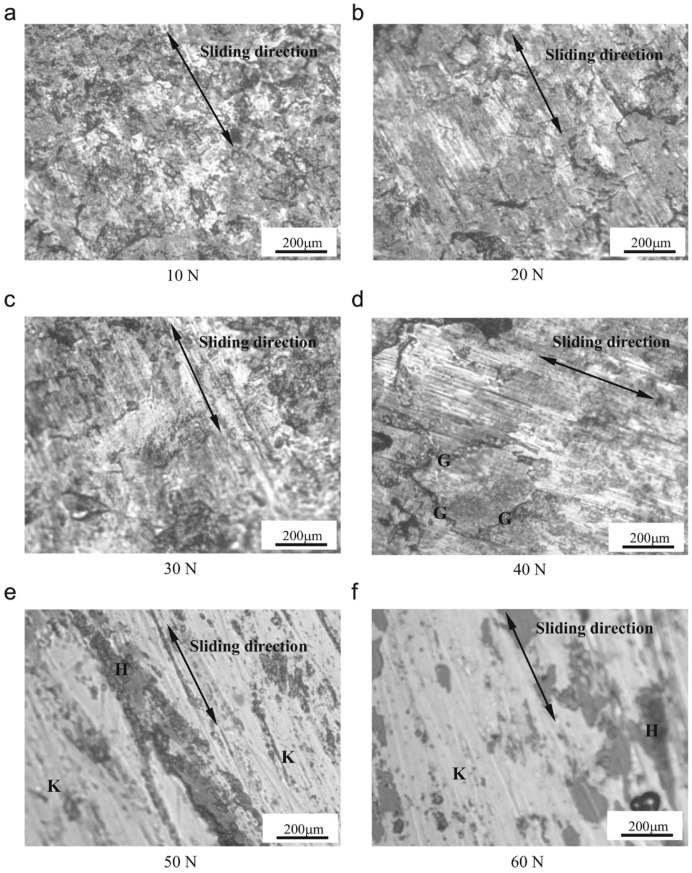
(**a**–**f**) Optical microscopy images showing wear surface morphologies of high-energy shot-peened medium carbon steel surfaces under various applied loads [36]. (**a**–**c**) Formation of shallow grooves and small worn tracks after plowing action and micro-cutting. (**d**) Formation of fatigue cracks (marked by ‘G’). (**e**,**f**) Formation of deep spalling tracks (marked by ‘H’) due to plastic removal and surface fatigue fracture of the deformed layer (marked by ‘K’), while simultaneously wearing out the harder nanocrystallized surface layer in the grey regions (‘H’).

In another research investigation, high-energy shot peening (HESP) using 8 mm diameter hardened ceramic balls was employed to develop a nanocrystalline surface layer on medium carbon steel, and the corresponding friction and wear behavior were analyzed and compared with an annealed counterpart [36]. The results [36] demonstrated that wear mass loss increased with higher applied loads for both the HESP-treated and annealed samples. Specifically, when the applied load was below 40 N, the wear resistance of the shot-peened steel outperformed that of its counterpart. However, when the applied load exceeded 40 N, the wear resistance of the shot-peened steel became inferior to that of its annealed counterpart [36]. This distinction is illustrated by the surface morphologies of the worn specimens shown in Figure 10. As depicted in Figure 10a–d, when the load was less than 40 N, surface damage occurred due to plowing and micro-cutting caused by abrasive particles dislodging from the alloy surface. The higher hardness and strength of the nanocrystallized surface layer, coupled with its residual compressive stress, inhibited the expansion of embryonic cracks, impeding fatigue crack development. However, at loads exceeding 40 N (Figure 10e,f), deep spalling tracks resulted from plastic removal and surface fatigue fracture because abrasive particles could penetrate more deeply into the surface [36].

**Figure 11:**
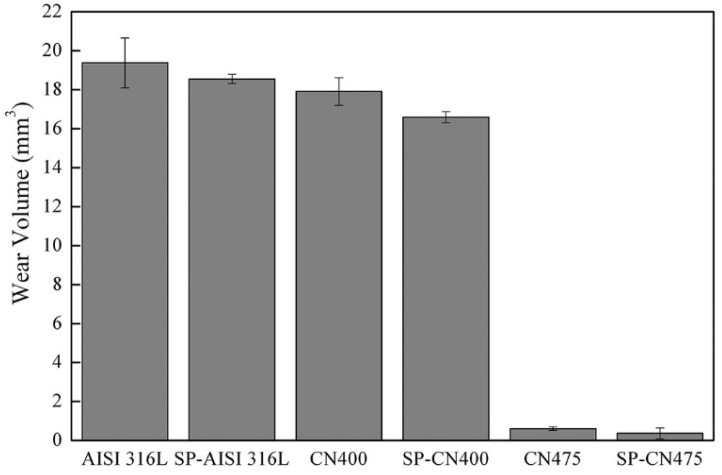
Wear loss after dry sliding wear experiments of AISI 316L stainless steel samples shot-peened (SP-AISI 316L), carburized, and then nitrided at 400 °C (CN400); shot-peened, carburized, and then nitrided at 400 °C (SP-CN400); carburized and then nitrided at 475 °C (CN475); and shot-peened, carburized, and then nitrided at 475 °C (SP-CN475) [37].

Post-surface treatments can also exert an influence on the wear characteristics of ferrous alloys. In a study conducted by Menezes et al. [37], 316L austenitic stainless steel underwent shot peening using stainless steel balls ranging from 0.09 mm to 0.20 mm in diameter. Subsequently, a sequential plasma treatment involving carburizing followed by nitriding was applied to 316L austenitic stainless steel at two different temperatures: 400 °C and 475 °C. This treatment resulted in the formation of distinct inner and outer layers, with the inner layer comprising carbon-enriched austenite and the outer layer consisting of nitrogen-expanded austenite. The surface hardening observed at 400 °C was attributed to the increased concentration of carbon and nitrogen within the solid solution. Conversely, at 475 °C, the surface hardening was primarily driven by the precipitation of chromium nitrides (CrN), facilitated by the presence of a carbon- and nitrogen-enriched solid solution. It was elucidated that the sequential plasma treatment performed on the shot-peened alloy at 475 °C yielded the highest level of wear resistance (Figure 11). This outcome was attributed to the formation of a thicker carbon-rich layer beneath the thinner but harder nitrogen-rich layer. This configuration provided enhanced mechanical support for the formation of oxides during wear, consequently improving wear performance [37].

In a recent study [38], 2205 duplex stainless steels subjected to shot peening using 0.3 mm stainless steel balls underwent post-surface polishing, and researchers investigated its wear behavior in simulated seawater. Notably, the shot-peened and post-surface polished samples demonstrated significantly lower wear loss compared to the untreated specimen. This improved wear resistance was attributed to the presence of strain-induced martensite, twins, and dislocations, which enhanced the material’s load-carrying capacity and reduced the depth of surface grooves, rendering the alloy more resistant to wear. In addition, the post-surface polished sample exhibited superior wear resistance due to the adverse impact of increased surface roughness caused by shot peening on the structural integrity of the as-received surface. This surface roughness contributed to higher wear rates in the as-received shot-peened specimen [38].

#### Nano-Scale Surface Peening

The nano-scale surface peening technique using 0.1–2.0 mm diameter shots, used for surface nanocrystallization, was employed by Ben Saada et al. [39] to modify the surface of 304L austenitic stainless steel. Subsequent investigation of the tribocorrosion behavior of the surface-treated ferrous alloy in a mixture of olive pomace and tap water filtrate under both continuous and intermittent sliding conditions revealed abrasive wear as the predominant mechanism governing the wear process in both scenarios. Specifically, during continuous sliding, the mechanical wear resistance was enhanced due to the high hardness and the presence of the nanocrystallized layer without significant changes in corrosion resistance. In contrast, during intermittent sliding, a top layer enriched with chromium (Cr) and molybdenum (Mo) containing ferrite grains contributed to the formation of the Cr- and Mo-rich corrosion film on the surface, resulting in improved corrosion resistance. Simultaneously, the work-hardened surface layer, developed after the surface treatment, played a role in boosting mechanical wear resistance [39].

In a subsequent research study conducted by Ben Saada [19], nanopeening treatment was applied to develop a nanocrystalline surface layer on martensitic stainless steel. The tribocorrosion performance of this alloy was then assessed in boric acid and lithium hydroxide solutions under both continuous and intermittent sliding conditions. Overall, the nanopeening treatment had a positive impact on the tribocorrosion resistance of the alloy. In both continuous and intermittent sliding scenarios, wear damage was primarily governed by adhesive and abrasive wear, although abrasive wear was more dominant in the nanopeened sample. Specifically, during intermittent sliding, mechanical wear was dominant due to the presence of hard debris produced from the repassivated layer. However, the nanopeened surface layer improved the mechanical resistance of the repassivated surface. On the other hand, nanopeening surface treatment improved both mechanical and corrosion resistance under continuous sliding conditions, ultimately leading to enhanced wear resistance [19].

### 4.3. Sandblasting

Sandblasting serves as another effective method for inducing surface nanocrystallization in ferrous alloys. In a study conducted on 304 stainless steel, sandblasting using silica particles (50–70 mesh) was employed to create a nanocrystalline surface layer with an average grain size of approximately 20 nm, followed by a subsequent annealing process [40]. The results of this investigation revealed that the mechanical properties of the sandblasted alloy surpassed those of the as-received alloy, primarily attributed to the high-density grain boundaries that impede dislocation movement. Furthermore, annealing further enhanced the mechanical characteristics of the sandblasted alloy by reducing dislocation density and refining grain boundaries with misorientations between adjacent grains [40].

The study also evaluated the wear and nano-wear resistance of the specimens [40], ranking them as follows (Table 3): annealed sandblasted 304 SS > sandblasted 304 SS > as-received 304 SS. In the wear tests, lower volume losses observed under corrosive wear conditions were associated with reduced frictional forces resulting from the lubricating effect of a NaCl solution. Considering the multiple factors influencing wear, such as hardness, adhesion, and elasticity, it was reported [40] that the sandblast-annealing treatment resulted in the highest wear resistance due to its superior mechanical properties, while the as-received sample exhibited the lowest wear resistance.

**Figure 12:**
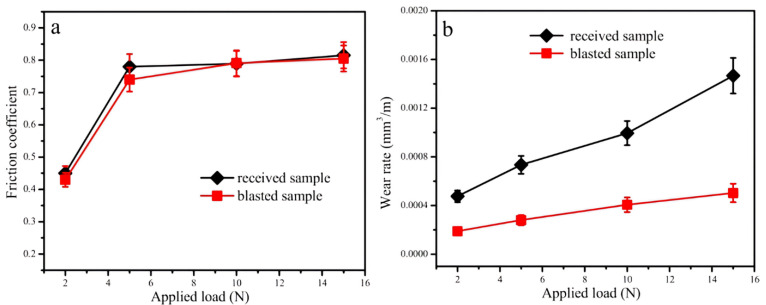
(**a**) Coefficient of friction and (**b**) wear rates of 1090 steel specimens under different applied loads [41].

In a subsequent study [41], the wear response of carbon steel that had undergone sandblasting to form a nanocrystalline surface layer, followed by annealing at 500 °C for 30 min, was investigated and compared with its as-received counterpart. The findings indicated that the coefficient of friction showed only slight variations with changes in grain size (Figure 12a). However, the wear rate exhibited a significant decrease in the surface-treated alloy compared to the untreated counterpart (Figure 12b).

The sandblasting process involved repeated shock loading at high speeds, resulting in an extremely high strain rate in the range of 10^3^–10^4^ s^−1^. This high strain rate not only significantly increased the dislocation density but also raised the carbon diffusion coefficient due to localized temperatures exceeding 1000 °C [41]. As a result, parts of the cementite phase dissolved into the nanostructured ferrite. Furthermore, a martensitic microstructure formed within the nanocrystalline surface layer during wear. This martensitic phase exhibited higher hardness than pearlite. The enhanced hardness of the sandblasted alloy, resulting from strain hardening and the presence of cementite/martensite phases, contributed to improved wear resistance. This shift in the wear mechanism was characterized by a transition from oxidation and adhesion to oxidation and plastic deformation [41].

### 4.4. Supersonic Fine Particle Bombardment (SFPB)

**Figure 13:**
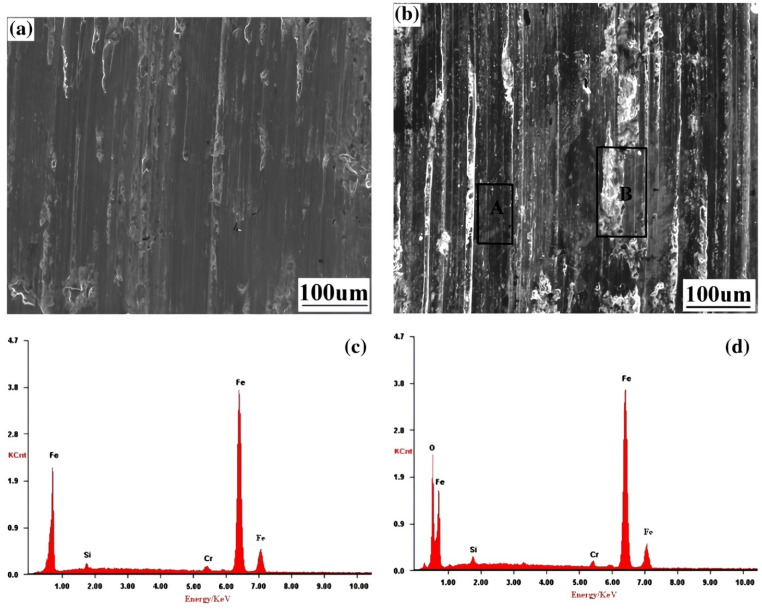
Surface morphologies associated with wear scars on SFPBed alloy steel specimens under loads (**a**) 3 N and (**b**) 9 N, and EDS area analysis in corresponding (**c**) region “A” and (**d**) area “B” [42].

Supersonic fine particle bombardment (SFPB) stands as another successful surface nanocrystallization (SN) technique utilizing stainless steel shots 0.4–0.6 mm in diameter to create a nanocrystalline surface layer of 25 µm thickness featuring grain sizes of approximately 16 nm on quenched and tempered chrome-silicon alloy steel [42]. Findings have shown that regardless of the surface treatment, wear volume loss increased with higher applied loads. However, it is noteworthy that the wear volume loss of the SFPBed sample consistently remained lower than that of the untreated alloy. Furthermore, as the applied loads increased, both SFPBed and untreated samples exhibited deeper and wider grooves (Figure 13) [42].

The predominant wear mechanism observed in the SFPBed alloy was primarily abrasive wear, inferred from Figure 13c,d. Notably, the “B” region in Figure 13d displayed a higher oxygen (O) content compared to the groove marked as “A” in Figure 13c [42]. This suggests increased surface activity in the SFPBed steel, resulting in the formation of a continuous oxide film. This film serves a dual purpose: protecting the surface and lubricating the alloy surface, thereby reducing friction and wear. During sliding wear, the continuous oxide film might break and penetrate the bulk material due to interactions with the ball and abrasive particles, resulting in lower O content within the grooves compared to the surrounding regions. On the other hand, for the untreated alloy, wear was governed by a combined action of adhesive and abrasive wear. This was due to its high plasticity, resulting in wear loss from the formation of parallel grooves caused by plowing, micro-cutting, and sticking. Plastic removal and spalling loss were more pronounced in the untreated alloy [42].

In simpler terms, abrasive wear resistance correlates with surface hardness, while higher alloy plasticity leads to more severe adhesive wear [42]. Therefore, the wear mechanism of the surface nanocrystallized steel was primarily abrasive wear, given its reduced tendency for plastic deformation/removal due to the higher hardness resulting from surface grain refinement. Additionally, it exhibited higher surface reactivity, fostering the development of an oxide film that could effectively lubricate the alloy surface [42].

In a subsequent study [43], supersonic fine particle bombardment (SFPB) was utilized to create a nanocrystalline surface layer on 1Cr18Ni9Ti austenitic stainless steel. The study revealed significantly lower wear mass losses in the SFPBed samples compared to the as-received stainless steel. In the as-received stainless steel, abrasive and adhesive wear dominated due to adhesion tearing, micro-cutting, and severe plastic deformation causing material transfer. In contrast, for the SFPBed stainless steel, the wear damage was attributed to a combination of fatigue, abrasive, and adhesive wear. SFPB enhanced the surface hardness while keeping the bulk material soft, thereby reducing plowing and adhesion due to the surface-hardened layer. In addition, the high chemical activity of the nanocrystalline layer facilitated the absorption of oxygen, promoting the formation of an iron oxide film. This oxide film acted as a lubricant, reducing direct contact between fresh metal surfaces. However, it is imperative to note that the high density of defects, such as dislocations, concentrated in the subsurface of the SFPBed stainless steel made it susceptible to crack initiation, leading to eventual spalling due to periodic friction stress [43].

Post-surface nanocrystallization indeed altered the wear resistance of ferrous alloys. Yang et al. [44] conducted a study to investigate the impact of vacuum carburization on the wear characteristics of a structural steel surface treated using 120 µm shot-pills under both dry and seawater conditions. Their research showed that the grain refinement occurring within the nanostructured surface layer provides additional diffusion paths during vacuum carburization, thereby improving the carburization rate and subsequently enhancing surface hardness. This enhancement in hardness resulted from refined carbides with a uniform distribution, which was produced through prolonged carburization, as well as lath martensite with a reduced amount of twinned martensite due to SFPB. These factors collectively contributed to the achievement of higher hardness levels, leading to superior wear resistance and lower friction coefficients under both dry and seawater conditions. The study [44] further demonstrated that under dry conditions, abrasive wear was the predominant mechanism responsible for wear damage, while under seawater conditions, wear was governed by plowing and corrosion attack.

Ding and Li’s recent investigation [45] also delved into the impact of post-surface modification on the wear resistance of 321 austenitic stainless steel that was SFPB-treated using 20–30 µm particles. They deposited a thin layer of FeS with exceptional anti-wear properties onto the resulting nanocrystalline surface layer. The study revealed that the untreated samples and those subjected to single-sulfurization treatment experienced severe abrasion. However, the introduction of SNC significantly enhanced wear resistance, and the presence of the compound-modified film clearly bolstered tribological properties. The outstanding wear resistance observed in the surface nanocrystallization- and sulfurizing-treated sample was attributed to two key factors: the increased hardness of the substrate and the augmented thickness, density, and uniformity of the sulfurized layer [45].

### 4.5. Fast Multiple Rotation Rolling (FMRR)

Fast multiple rotation rolling (FMRR) [46] stands out as another surface nanocrystallization technique applied to modify low-carbon steel surfaces. The steel subjected to FMRR exhibited improved wear resistance, characterized by a lower friction coefficient and reduced wear volume loss. This enhancement can be attributed to the formation of a hard nanocrystalline surface layer. During the initial 50 min of testing, the friction coefficient of the FMRR-treated sample gradually increased. This rise was due to a continuous hard nanocrystalline layer on the surface, and the chemically active surface layer facilitating the formation of an oxide film. This oxide film significantly reduced friction and direct contact between fresh metal surfaces. However, after the initial 50 min, the friction coefficient of the FMRR-treated specimens increased rapidly, indicating that the nanostructured layer had been completely worn away. In summary, the total wear mass loss of the FMRR-treated sample was lower than that of the untreated one, underscoring the improved wear resistance achieved through FMRR surface modification [46].

### 4.6. High-Speed Pounding (HSP)

**Figure 14:**
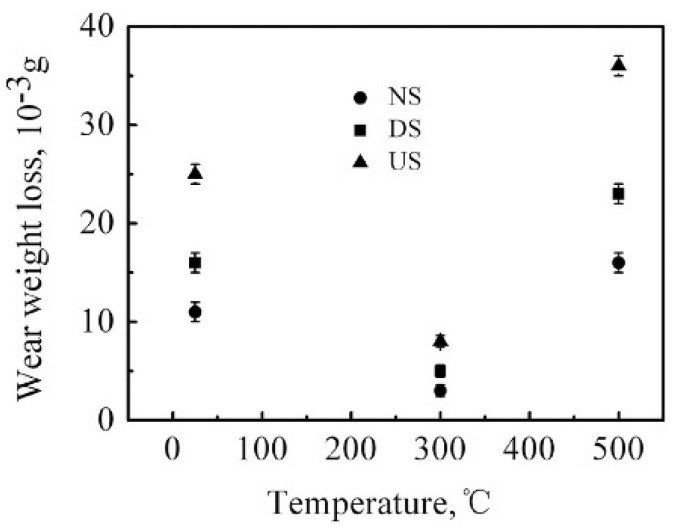
Wear mass loss of surface nanocrystallized (NS), deformed (DS) with the same hardness as NS, and untreated (US) Hadfield steel specimens at different temperatures [47].

High-speed pounding (HSP) is another surface nanocrystallization (SNC) technique that is effectively applied to treat Hadfield steel surfaces. The resulting samples were then subjected to wear tests at three different temperatures [47]. As depicted in Figure 14, the wear mass loss of the surface nanocrystallized (NS) sample was lower than that of the deformed (DS) sample, despite both having the same hardness as the NS sample, as well as the untreated (US) specimen. Moreover, the wear weight loss at 300 °C was the lowest among these samples. Conversely, all samples exhibited the highest wear mass losses at 500 °C, which were even greater than those observed at 25 °C. The study reported that at 25 °C, no oxide formation was observed on the untreated and deformed samples. However, the presence of an oxide film on the wear track of the surface nanocrystallized specimen altered the main wear mechanism from adhesive to slightly abrasive wear. At elevated temperatures of 300 °C and 500 °C, a continuous oxide film developed on the contact area during wear, which effectively reduced mechanical damage by preventing direct metal-to-metal contact. However, the thermal softening effect was more pronounced at 500 °C compared to 300 °C. As a result, the underlying substrate was unable to adequately support the oxide film, leading to the accelerated delamination of the oxide layer. This explains why the wear mass loss for the same samples was least at 300 °C and highest at 500 °C [47].

### 4.7. Ultrasonic Impact Treatment (UIT)

**Figure 15:**
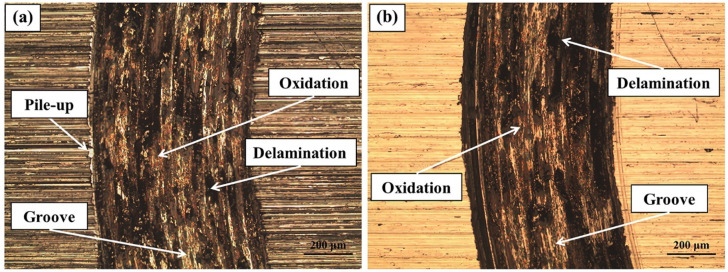
Optical microscopy images taken from wear tracks on (**a**) untreated and (**b**) three passes-treated stainless steel specimens [48].

Ultrasonic impact treatment (UIT) represents another ultrasonic-based technique employed for the surface nanocrystallization of ferrous alloys. Although the specific study [48] did not provide information on the grain size and thickness of the nanocrystalline deformed region within the surface-treated stainless steel alloy, it inferred the formation of a surface nanocrystallized layer primarily through hardness measurements. It is worth noting that despite similar coefficients of friction between the untreated and surface-treated samples, the wear loss of the surface-treated sample was notably lower than that of the untreated one. This discrepancy may be attributed to the surface nanocrystallization (SNC)-induced hardening and the transformation of austenite into the hard α and ε martensitic phases. Moreover, both specimens exhibited dominant wear mechanisms, including abrasive, adhesive, and oxidative wear (Figure 15). It is pertinent to mention that the surface-treated alloy did not exhibit pile-up along the edges of the wear track, likely due to its higher hardness (Figure 15) [48].

In another study [49], the surface of 316L austenitic stainless steel underwent UCFT treatment, and the treated alloy was subjected to wear testing under various lubricated conditions. The surface-treated and untreated stainless steel specimens were tested using the synthetic oil Polyalphaolefin (PAO4) both with and without the addition of 10 wt.% molybdenum dithiocarbamate (MoDTC) and zinc dialkyldithio-phosphate (ZDDP) additives. The results showed that UCFT treatment significantly improved the wear resistance of the alloy, as evidenced by reductions in both friction coefficient and wear rate. Furthermore, the presence of MoDTC and ZDDP additives in PAO4 further enhanced the tribological properties. Specifically, the lowest friction coefficient was achieved when MoDTC was used as a lubricant, while the lowest wear loss was observed when ZDDP was added to the lubrication system [49].

**Figure 16:**
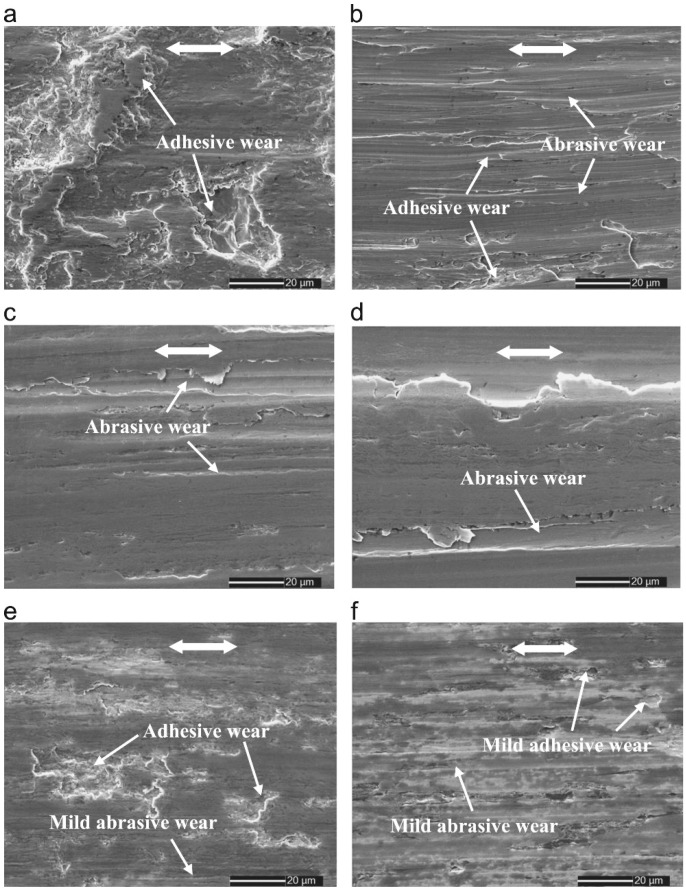
SEM images of the worn 316L stainless steel surfaces: (**a**) untreated surface, PAO4; (**b**) UCFT surface, PAO4; (**c**) untreated surface, MoDTC; (**d**) UCFT surface, MoDTC; (**e**) untreated surface, ZDDP; (**f**) UCFT surface, ZDDP [49].

In general, the data presented in Figure 16 indicates that surface nanocrystallization can markedly reduce adhesive wear, particularly when additives are introduced into the lubricating oil. In the case of additive-free PAO4 lubrication [49], the higher number of dimples on the surface nanocrystallized alloy, which resulted in a rougher surface, served as a reservoir for the oil. This helped maintain a thin oil layer between the rubbing surfaces, which in turn reduced friction and improved wear resistance (Figure 16a,b). Moreover, the higher hardness of the UCFT-treated sample served a role in reducing adhesive wear, further enhancing its wear resistance [49].

When MoDTC was added to PAO4, the formation of a tribofilm containing MoS_2_ (molybdenum disulfide) significantly reduced wear mass loss and the coefficient of friction (Figure 16c,d). It was found [49] that the MoS_2_/MoO_3_ ratio in the tribofilm determined the wear behavior, with higher ratios leading to better wear resistance. The UCFT-treated sample had a higher MoS_2_/MoO_3_ ratio due to its greater surface reactivity, which contributed to its improved wear resistance. On the other hand, when ZDDP was added to the PAO4 lubricant, zinc phosphate glasses were formed at the interface of the rubbing surface (Figure 16e,f). These glasses acted as a protective layer, reducing wear damage. The higher surface activity of the UCFT-treated sample also played a role in the formation of shorter chain phosphates with better mechanical properties on the surface, further enhancing wear resistance in the presence of ZDDP [49].

In a subsequent study [50], this technique was utilized to modify the surface of an iron-based alloy deposited on a low-carbon steel substrate. The study assessed the wear resistance under lubricated conditions and compared it to the as-deposited coating. The enhanced hardness observed in the surface-treated coating was attributed to grain refinement and the formation of a martensite phase through deformation. This, in turn, led to an improvement in the coating’s wear resistance, causing a shift in the wear mechanism from adhesive wear for the as-deposited coating to abrasive wear for the surface-treated one [50].

### 4.8. Ultrasonic Surface Rolling (USR) Process

Ultrasonic surface rolling (USR), an ultrasonic-based surface nanocrystallization technique, was applied to 316L stainless steel in a study [51]. This process produced a nanostructured surface layer with a thickness of 15 μm, exhibiting higher hardness than the as-received counterpart. Consequently, the surface-treated steel demonstrated superior wear performance when subjected to severe abrasive wear under various experimental conditions. At both low and high sliding speeds, the wear mechanism for the as-received alloy primarily involved oxidation and adhesive wear. However, in the surface-treated alloy, abrasive wear became dominant. Notably, at low sliding speeds, the presence of an oxide film on both the sample and the slider surface heightened the influence of oxidation wear, while at high wear test speeds, direct metal-to-metal contact became the controlling factor in the wear mechanism [51].

The high-frequency impacting and rolling (HFIR) technique has also been employed to create a nanocrystallized surface layer on 40Cr steel [52]. The process of surface nanocrystallization was achieved through the formation of dislocation tangles, the transformation of subgrains into nanocrystals, and the breakage of pearlite. Although the nanostructured surface layer exhibited enhanced hardness, there was no significant change in the friction coefficient when compared to the as-received specimen. However, the wear mass loss of the nanocrystalline surface layer was markedly lower than that of the as-received sample. With an increase in the applied load during wear testing, the dominant wear mechanism for the as-received sample shifted from abrasive wear to fatigue wear. In contrast, for the nanocrystalline surface layer, the dominant wear mechanisms remained consistent under all applied loads, primarily involving oxidation wear and adhesive wear. It was elucidated that the presence of a hard nanocrystalline structure, along with compressive residual stress, effectively prevented crack initiation, while the coarse-grain structure acted as a barrier to crack propagation [52]. This explains why fatigue wear did not occur in the nanocrystalline surface layer.

In a later study [53], the USR processing method was employed to treat the surface of high-carbon high-chromium steel, causing the fragmentation and dissolution of long rod-shaped primary carbides. Furthermore, continuous dynamic recrystallization during the USR process resulted in the formation of equiaxed grains. Interestingly, both of these phenomena observed during USR processing somewhat limited further enhancements in hardness. As a consequence, the wear test results revealed that the wear volume of the USR-treated steel was similar to that of the untreated specimen. However, the refined microstructure prevented the initiation and propagation of cracks, ultimately improving wear resistance [53].

In a recent study [54], surface nanocrystallization of M50-bearing steel was achieved using USR processing. The investigation aimed to clarify the influence of USR processing variables on surface performance, with the observed order of importance being feed rate > load > rolling time. The results of the study showed that the wear rate and coefficient of friction of the USR-treated specimen were lower than those of the untreated counterpart. This improvement was attributed to the formation of a smoother tribofilm on the surface, which led to a shift in the wear mechanism from abrasive/adhesive wear for the untreated sample to slight adhesive wear for the surface-treated alloy [54].

### 4.9. Surface Mechanical Rolling Treatment (SMRT)

A nanostructured surface layer was successfully achieved on 316L austenitic stainless steel using surface mechanical rolling treatment, and its tribocorrosion resistance was subsequently assessed in both pure water and a 1 M HCl solution [55]. The study [55] revealed that corrosion accelerated wear loss, primarily through delamination. However, the nanocrystallized surface layer effectively inhibited delamination in the corrosive solution due to its harder surface and the presence of residual stress. It also reduced material loss attributed to abrasive wear in pure water.

## 5. General Discussion

Table 4 provides a comprehensive overview of various ferrous alloy grades, their corresponding surface properties, characteristics of nanocrystalline surface layers, and the experimental conditions for wear tests. Notably, approximately half of the studies in this summary have primarily explored the influence of SNC on the wear behavior of stainless steels, while others focused on carbon steels. However, not all studies in Table 4 provide detailed information on crucial aspects like grain size, layer thickness, and surface roughness. Typically, in the case of SNC-treated alloys, the grain size was consistently found to be lower than 40 nm, while the thickness of the surface-treated layer generally remained under 150 μm for ferrous materials. An additional contributing factor, surface roughness, has also not been uniformly reported across all research studies. The change in surface roughness appears to be highly dependent on the specific SNC technology employed for surface treatment. It can either increase, decrease, or have no significant impact on surface roughness, as observed in different studies in Table 4.

The findings summarized in Table 4 consistently demonstrate that surface nanocrystallization (SNC), and in some cases, SNC followed by post-treatment, has consistently led to an enhancement in the wear resistance of ferrous alloys across various experimental conditions. Notably, none of the reviewed studies report inferior wear properties for SNC-treated ferrous alloys, indicating the robustness and effectiveness of this approach.

Table 4 suggests that nano-scale surface peening, sandblasting, supersonic fine particle bombardment, fast multiple rotation rolling, ultrasonic surface rolling, and surface mechanical rolling treatment enhance the abrasive wear resistance of ferrous alloys. When considering plowing (occurring in ductile materials due to the passage of asperities or abrasive particles of a hard surface on a softer one) and micro-cutting (also occurring in ductile materials and resulting from the cutting of material ahead of asperities or abrasive particles, leading to chip formation) as subcategories of abrasive wear, surface mechanical attrition treatment, shot peening, and supersonic fine particle bombardment have proven effective in reducing the level of plowing and micro-cutting in Fe-based materials.

Moreover, adhesive wear in steels can be significantly mitigated by employing sandblasting, which shifts the wear mechanism from oxidation and adhesion to oxidation and plastic deformation. Similarly, supersonic fine particle bombardment, high-speed pounding, and ultrasonic impact treatment enhance resistance to adhesive wear by inducing a transition from adhesive to abrasive wear. Likewise, tribocorrosion resistance can be enhanced by utilizing surface mechanical attrition treatment, shot peening, nano-scale surface peening, and sandblasting technologies. Finally, surface mechanical attrition treatment, shot peening, high-frequency impacting, and rolling have been identified as effective measures for reducing fatigue wear.

This underlines SNC as a powerful tool for improving the wear resistance of ferrous alloys. Depending on factors such as the specific alloy, the SNC technology employed, and any subsequent post-treatment, the processing parameters can be optimized to achieve the desired level of wear resistance. The resulting wear resistance can vary and is closely related to factors like surface hardness and the development of a tribolayer, which will be further discussed in the following section.

**Table 4:** Summary of surface nanocrystallized ferrous alloys, their surface properties, and wear behavior. *R_a_* stands for the alloy’s surface roughness. Alloys are marked “*SNC*” for surface nanocrystallized and “*UT*” for untreated.

Surface Treatment Method	Material	Grain Size (Deformed Region Thickness)	$\frac{{R_{a}}_{SNC}(µm)}{{R_{a}}_{UT}(µm)}$	Wear Test (Condition)	General Comments	Ref.
Surface Mechanical Attrition Treatment	Low-carbon steel	10–20 nm (10 µm)	$\frac{0.59}{0.67}$	Reciprocating sliding wear test (dry)	Enhanced wear resistance and lower coefficient of friction due to surface hardening.	[30]
	304 stainless steel	Not reported (Not reported)	$\frac{0.33-0.81}{0.07}$	Pin-on-disk test (unlubricated and lubricated)	The wear resistance of the SMATed alloy demonstrated an improvement under lubricated conditions, whereas it remained relatively unchanged under dry conditions.	[31]
	304 stainless steel	Not reported (Not reported)	$\frac{0.38}{0.04}$	Tribocorrosion test (NaCl solution)	The tribocorrosion resistance of SMATed SS was found to be better than that of the untreated alloy under different overpotentials.	[32]
	321 stainless steel	18 nm (Not reported)	$\frac{1.00}{0.03}$	Reciprocating ball-on-disc (dry)	Improved wear resistance of nitrided SMATed SS compared to untreated counterpart.	[33]
Shot peening	Hadfield steel	11.1–17.4 nm (100 µm)	Not reported	two-body and three-body abrasive wear tests (dry)	Surface nanocrystallization improved the three-body wear resistance of the alloy compared to its untreated counterpart, but it did not alter its two-body wear resistance. Light impact load was also found to be beneficial in improving wear resistance.	[34,35]
	Medium carbon steel	20 nm (30 µm)	$\frac{1.12}{2.44}$	Ring-on-disc test (dry)	The wear resistance of shot-peened alloy was enhanced compared to its annealed counterpart, primarily due to the higher hardness and presence of residual stress, when the applied load remained below a specific threshold.	[36]
	316L stainless steel	Not reported (Not reported)	$\frac{1-2}{2}$	Ball-on-disk test (dry)	The highest level of wear resistance was achieved with the sequentially plasma-treated stainless steel, first carburized and then nitrided at a higher temperature.	[37]
	2205 stainless steel	Not reported (30 µm)	$\frac{6.43}{0.021}$	Ball-on-disk test (simulated seawater)	Shot peening effectively enhanced the wear resistance of the alloy, and the subsequent surface polishing further improved this wear resistance by mitigating the negative effects of high surface roughness.	[38]
Nano-scale surface peening	304L stainless steel	Not reported (150 µm)	Polished	Pin-on-disk tribocorrosion test (corrosive solution)	The tribocorrosion resistance of the surface-treated SS was improved under all experimental conditions.	[39]
	420 stainless steel	Not reported (110 µm)	Not reported	Pin-on-disk tribocorrosion test (corrosive solution)	The tribocorrosion resistance of nanopeened SS was superior to that of its untreated counterpart under different sliding conditions.	[19]
Sandblasting	304 stainless steel	20 nm (70 µm)	Not reported	Ball-on-disk sliding wear test/Scratch test (dry and corrosive solution)	Annealing contributed to an enhancement in the wear resistance of sandblasted stainless steel (SS) by improving its mechanical properties.	[40]
	1090 steel	78 nm (30 µm)	Not reported	Ball-on-disk sliding wear test (dry)	Sandblasting led to phase transformation combined with increased surface hardness, which improved the wear resistance and altered the wear mechanism.	[41]
Supersonic Fine Particles Bombardment	Chrome-silicon alloy steel	16 nm (25 µm)	Not reported	Ball-on-disc wear test (dry)	The transition from a combination of abrasive and adhesive wear in the untreated alloy to abrasive wear in the surface-treated one was a key factor contributing to enhanced wear resistance of the surface nanocrystallized alloy.	[42]
	1Cr18Ni9Ti stainless steel	30 nm (10 µm)	Not reported	Ball-on-disc wear test (dry)	The improved wear resistance of SFPBed stainless steel was attributed to two factors: the higher hardness of the surface layer and the lubrication effect of the oxide film formed due to the increased chemical activity of the surface.	[43]
	18Cr2Ni4WA steel	16.6–45.5 nm (53–74 µm)	Not reported	Ball-on-disc wear test (dry and seawater)	Carburization further boosted the positive influence of SNC on wear resistance.	[44]
	321 stainless steel	21 nm (Not reported)	Not reported	Ball-on-disc wear test (dry)	The addition of the FeS deposited layer onto the SFPB-treated stainless steel further enhanced its wear resistance.	[45]
Fast multiple rotation rolling	Low carbon steel	8–18 nm (30 µm)	$\frac{0.47}{0.16}$	Ball-on-disk test (dry)	The wear resistance of FMRR-treated steel was superior to that of the untreated steel.	[46]
High-speed pounding	Hadfield steel	25 nm (Not reported)	Not reported	Pin-on-disc test (dry)	The nanocrystallized surface enhanced wear resistance under all experimental conditions. The highest wear resistance was observed at 300 °C, while the lowest was at 500 °C, and wear resistance was at a moderate level at 25 °C.	[47]
Ultrasonic impact treatment (Ultrasonic cold forging technology)	SUS301 stainless steel	19 nm (150 µm)	$\frac{0.69}{0.11}$	Pin-on-disk wear test (dry)	Multiple UIT enhanced the wear resistance of austenitic SS, although it had no noticeable effect on the friction coefficient.	[48]
	316L stainless steel	19 nm (150 µm)	$\frac{0.016}{0.004}$	SRV reciprocating wear test (lubricated)	UCFT treatment significantly improved wear resistance under lubricated conditions. Moreover, the presence of additives in the lubricating oil further enhanced the wear performance due to the formation of a protective tribofilm.	[49]
	Iron-based alloy	100 nm (34 µm)	Not reported	Ring-on-block wear test (lubricated)	The wear properties of the iron-based deposited coating were improved compared to the as-deposited counterpart. The wear mechanism also changed from adhesive to abrasive wear after surface treatment.	[50]
Ultrasonic surface rolling (High-frequency impacting and rolling)	316L austenitic stainless steel	6–15 nm (15 µm)	$\frac{0.04}{0.80}$	Ring-on-block wear test (dry)	Wear resistance USR-treated alloy was enhanced under both low and high sliding speeds.	[51]
	40Cr steel	6.2 nm (80 µm)	Not reported	Pin-on-disk wear test (dry)	The HFIR process significantly improved the wear resistance of the alloy to the extent that it effectively prevented fatigue wear, even under high applied loads.	[52]
	X210CrW12 steel	40 nm (100 µm)	$\frac{0.21}{0.18-0.27}$	SRV reciprocating wear test (dry)	The wear volume loss of untreated and surface-treated samples were similar.	[53]
	8Cr4Mo4V bearing steel	Not reported (24 µm)	$\frac{0.15}{0.47}$	Ball-on-plate test (dry)	The wear properties were improved for surface-treated samples. The impact of USR-treatment parameters on the surface performance was also analyzed.	[54]
Surface mechanical rolling treatment	316L austenitic stainless steel	30 nm (200 µm)	$\frac{0.12}{Not\;reported}$	Crossed cylinder contact tribocorrosion test (corrosive solution)	The tribocorrosion resistance in both pure water and HCl solution was enhanced after surface treatment, which significantly reduced delamination.	[55]

## 6. Mechanisms for Enhanced Wear Resistance of SNC-Treated Steels

### 6.1. Surface Hardening

Surface hardness, a key outcome of *SNC*, plays a pivotal role in enhancing the wear resistance of ferrous alloys. Wear loss tends to inversely correlate with hardness, as described by Archard’s wear equation [41]:

(1) $$V=\;K\frac{L\times S}{H}$$

where the variables *V* (volume loss), *H* (hardness), *K* (wear friction coefficient), *S* (sliding distance), and *L* (normal load) are used to analyze the impact of surface hardness on wear rate [41]. The coefficient of friction is directly proportional to the frictional force under the same load. If the plastically deformed region beneath the asperity approaches the size of the actual contact area, then *K* denotes a ratio of worn volume to the plastically deformed region. In cases of adhesive wear, *K* serves as a loose indicator of the likelihood that an asperity adhesive junction results in a wear particle. Adhesive wear occurs when asperities come into contact under high local pressures, sometimes leading to weld formation that may be stronger than the bulk asperity, owing to the cohesive strength of the softer material being lower than the interfacial strength. In cases of abrasive wear, where the material of the asperity is harder than the surface material it plows through, a simplified plowing model yields *K* = tan ϑ/π, where ϑ represents the cutting angle. For adhesive wear, *K* typically ranges from approximately 10^−4^ to 10^−3^, while for abrasive wear, *K* is typically around 10^−1^ [56].

During wear testing, the frictional force is contingent upon the force necessary to plastically deform the surface [46]. When surfaces, as seen in *SNC*-treated alloys, are harder, they resist deeper indentation by the ball or pin tip during wear [46]. As a result, the force required to plastically deform the surface decreases [46], contributing significantly to the improvement of wear resistance [46].

#### 6.1.1. Grain Refinement

Generally, it has been reported [40,41,50,51,52,55] that the heightened hardness of surface nanocrystallized ferrous alloy resulting from its surface grain refinement is identified as the primary reason for its superior wear resistance. Additionally, this enhanced surface hardness achieved through SNC has proved beneficial in significantly improving the tribocorrosion resistance of ferrous metallic materials [32,55].

Surface severe plastic deformation induces a grain refinement mechanism [39]. The process begins with nano-scale surface peening, involving repeated high-energy impacts at high rates on the specimen surface. These impacts generate and increase the number of dislocations within the material. Ultimately, these dislocations either annihilate or rearrange themselves, forming small-angle grain boundaries that separate individual crystals, thereby leading to grain refinement. The refined microstructure of the surface layer results in an increase in hardness, following the Hall–Petch relationship [48]:

(2) $$H=H_{0}+\frac{K}{\sqrt{d}}$$

Here, *H* represents the material’s hardness, *K* is a material-specific constant that tends to increase with a higher Taylor factor, *H*_0_ is a constant related to the method of hardness measurement, and d represents the mean grain size. Therefore, when the grain size of the surface layer is reduced through *SNC*, it leads to surface hardening following the principles outlined in the Hall–Petch relationship [48]. Consequently, the formation of a nanocrystalline surface layer on *SNC*-treated Fe-based alloy increases surface hardness, leading to a reduced degree of plowing [30,31,43] and micro-cutting [30,34,43], as well as a decreased degree of plastic removal [30,31,42] and surface fatigue fracture [30].

#### 6.1.2. Strain-Induced Phase Transformation

Severe plastic deformation of the surface can induce significant strain rates, ultimately leading to phase transformation [41]. Given the local surface temperature potentially exceeding 1000 °C and the exceedingly high strain rate levels, typically falling within the range of 10^3^–10^4^ s^−1^, phase transformation becomes highly probable [41].

As elucidated in the introduction section, steels possess the capability to undergo solid-state phase transformations, leading to microstructural evolution. Within steels’ microstructures, certain phases play a crucial role in enhancing the wear resistance of the alloy surface, consequently improving overall wear performance. Notably, martensitic microstructure and nanostructured ferrite are regarded as favorable phases with high mechanical properties, thereby enhancing the wear behavior of SNC-treated steels. In this context, [41] has demonstrated that sandblasting applied to carbon steel resulted in the dissolution of cementite into nanostructured ferrite [41]. Simultaneously, wear contributed to the transformation of ferrite into martensite, characterized by higher hardness compared to pearlite, ultimately leading to improved wear performance [41]. It has also been found [38,48,50] that hard deformation-induced martensite increases the hardness of Fe-based alloys, thereby enhancing their wear resistance.

#### 6.1.3. Plasma Treatment

Plasma treatment-induced hardening has proven advantageous in enhancing the wear resistance of surface nanocrystallized Fe-based materials. In a particular case [37], the key factor contributing to this improvement in the wear resistance of ferrous alloy was the formation of a relatively thick carburized inner layer, which was overlaid by a thinner but harder nitrided layer enriched with chromium nitrides. This dual-layer structure enhanced wear resistance by providing crucial support for the formation of an oxide film during wear, effectively mitigating wear-related damage [37]. In another case, the increased hardness and improved load-bearing capacity resulting from the thicker nitrided layer and the gradual gradient in the hardness profile of the SNC-treated alloy subjected to plasma nitriding (with a thicker S-phase layer and gradient nitrogen diffusion layer) were identified as key factors significantly enhancing wear resistance [33].

### 6.2. Tribofilm Formation

The formation of tribofilm has been observed in both fluid lubrication and dry friction. This film plays a significant role in enhancing anti-friction and wear resistance in tribosystems. The effectiveness of a tribofilm is determined not only by the properties of the film-forming substance but also by the physical and chemical processes occurring on the surface during friction [57]. While thermal effects have traditionally been considered the primary factor in tribofilm formation, recent studies indicate that mechanical and electrical effects can also stimulate tribochemical reactions, thereby enhancing anti-wear and friction-reduction performance [58].

**Figure 17:**
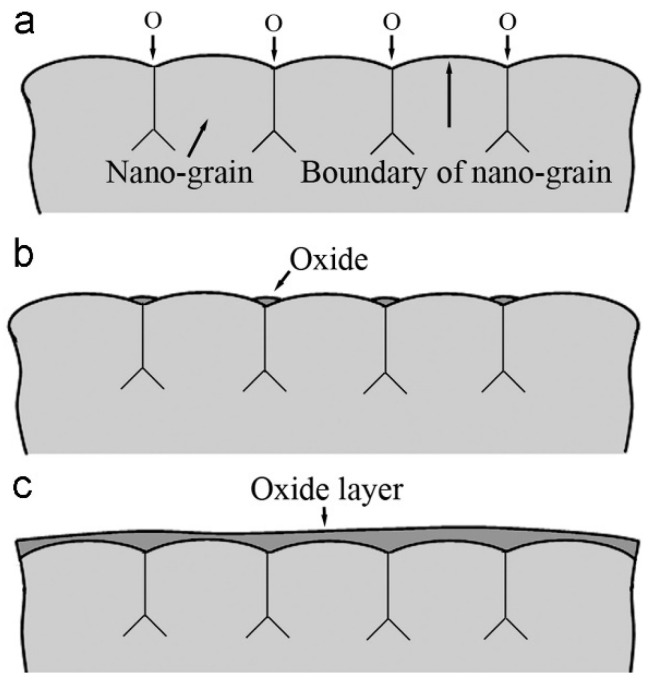
(**a**–**c**) Schematic representation of oxide film formation on the nanocrystallized alloy surface [47]. (**a**) Absorption of oxygen on the wear surface of SNC-treated alloy. (**b**) Preferential development of oxides at grain boundaries. (**c**) Gradual increase in oxides due to enhanced surface activity, leading to the formation of an oxide layer through frictional processes and/or increased ambient temperature.

During the wear process under dry conditions, oxygen is adsorbed onto the surface of the surface nanocrystallized alloy, specifically at the grain boundaries (Figure 17a). These grain boundaries exhibit higher chemical activity and contain more defects due to the irregular orientation of atoms’ arrangements compared to the inner grain structure. Consequently, oxides begin to form at these grain boundaries whose density is significantly higher within the nanostructured surface layer (Figure 17b). This process promotes the gradual accumulation of oxide layers, which eventually coalesce to form a continuous oxide film on the surface (Figure 17c) [47]. This continuous oxide film serves a dual role in the surface-treated ferrous alloy [42,43,46,54]. Firstly, it modifies the wear mechanism, primarily shifting it towards abrasive wear. Secondly, it acts as a lubricant, effectively lowering the coefficient of friction and wear loss. Additionally, it reduces direct contact between fresh metal surfaces, further contributing to improved wear resistance [42,43,46,54].

Under lubricated conditions, the higher surface reactivity of a nanocrystalline surface layer, attributed to the presence of nanograins and a high density of grain boundaries, coupled with the lower atomic density at these grain boundaries, can facilitate the formation of reactive films [49]. This contributes to a reduction in the friction coefficient and a decrease in the wear rate since the developed tribofilm prevents direct contact between the rubbing surfaces [49].

For example, in a study [49], stainless steel treated with UCFT to create a nanocrystalline surface layer exhibited improved wear resistance under lubricated conditions, especially in the presence of additives that promote the formation of a tribofilm on the surface. The heightened surface reactivity of the UCFT-treated specimen played a crucial role in improving the formation of this tribofilm (MoS_2_/MoO_3_ and zinc phosphate glasses), ultimately resulting in higher wear resistance under lubricated conditions [49].

In addition, the presence of a composite surface layer composed of a thin FeS film on the surface-nanocrystallized 321 stainless steel had a pronounced effect in reducing both the wear rate and friction coefficient [45]. This combination of a soft anti-wear surface overlaying a hard substrate effectively mitigated cutting and adhesion effects, contributing to improved tribological performance [45].

## 7. Conclusions and Future Directions

**Figure 18:**
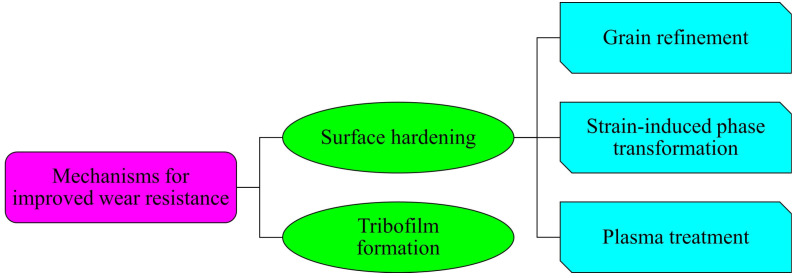
Mechanisms dictating improved wear resistance of SNC-treated ferrous alloys.

This paper presents an exploration of the impact of surface nanocrystallization treatment on the wear behavior of ferrous alloys. It demonstrated how *SNC* technologies can generate a nanocrystalline surface layer of approximately 150 μm thick, featuring grain sizes below 40 nm. Surface roughness in *SNC*-treated alloys was found to vary depending on the applied method. Importantly, all SNC-treated alloys exhibited significantly improved wear resistance compared to their untreated counterparts, highlighting the substantial role of *SNC* in enhancing the wear resistance of Fe-based materials. The predominant mechanisms governing improved wear resistance in *SNC*-modified Fe-based materials are summarized in Figure 18. Enhanced hardness resulting from *SNC* treatment and the formation of a tribolayer due to increased surface activity significantly contribute to the improved wear performance of these alloys. Consequently, *SNC* technologies emerge as promising approaches for enhancing the wear resistance of Fe-based alloys.

The challenges and future directions that highlight the research gaps in the current literature are presented as follows:

(1) Surface roughness in *SNC*-treated alloys remains inadequately explored. A comprehensive study is needed to investigate the influence of treatment variables on surface finish and its corresponding effect on the tribological behavior of surface nanocrystallized alloys.
(2) More detailed investigations into the wear mechanisms of surface nanocrystallized alloys are recommended. Current literature lacks comprehensive examinations of the wear mechanisms governing the degradation of metallic materials.
(3) Additive manufacturing (AM) technologies have found a unique niche for themselves in the production of near-net-shaped components with intricate designs. However, the resolution of components produced using AM methods, such as wire arc additive manufacturing (WAAM), can sometimes be unsatisfactory [59]. In this context, *SNC* treatments can be employed not only to enhance the surface finish of these parts [60] but also to improve the mechanical properties of the surface layer. This has the potential to facilitate the production of wear-resistant metallic components, which can then be subjected to wear behavior studies and compared to conventional parts.
(4) While indirect surface nanocrystallization techniques, such as cavitation peening and pulsed-high energy density plasma, have been utilized to modify the corrosion properties of ferrous alloys, they remain unexplored for anti-wear purposes in these materials. Thoroughly investigating the effects of these indirect methods on wear characteristics in ferrous materials is suggested.
(5) Combining different *SNC* techniques for surface modification of ferrous alloys could yield intriguing synergistic effects due to their unique characteristics. For example, the levels of hardness and roughness induced by each *SNC* technique may vary. Consequently, investigating the combined influence of multiple methods on wear responses compared to individual methods in ferrous alloys would be beneficial.
(6) To date, there has been no examination of the in situ wear behavior of *SNC*-treated alloys. It is essential to explore how nanocrystallized surface layers behave under in-situ microscopic observations, coupled with in situ surface analysis. This approach would provide a deeper understanding of the wear mechanisms at play in ferrous alloys.
(7) The impact of large grain boundary surface area per unit volume on the thermal stability of *SNC*-treated steels remains unexplored. Investigating the potential recrystallization and grain growth of nanograin structures during high-velocity sliding and its high heat generation effect is essential.
(8) The *SNC* treatment methods described in the present paper may yield a gradient distribution of grain sizes on the surface. Researchers are encouraged to delve deeper into the effects of this grain size gradient on the material’s wear properties.

## Figures and Tables

**Table 1:** Definitions of the most-accepted wear mechanisms [4].

Wear Mechanism	Description
Adhesive wear	Adhesive wear occurs when the asperities in the interface adhere to each other. These contact points can be separated as the surfaces move, potentially causing a fragment to break away from one surface and adhere to the other.
Abrasive wear	“Three-body abrasive wear” takes place when hard particles are present between the rubbing surfaces, resulting in material removal from both surfaces, or the embedding of these particles in the softer surface, which leads to material loss in the counter body. On the other hand, “two-body abrasive wear” happens when the hard surface’s asperities plow into the soft matrix of the counter body, creating debris particles. In practical scenarios, the most common type of wear often incorporates elements of both two-body and three-body abrasive wear.
Fatigue and fretting wear	Fatigue wear involves the cyclic growth of microcracks on the surface, which leads to the detachment of wear particles. As a result, the repetitive loading weakens the material’s surface. Fretting wear is defined as repeated cyclic rubbing between two surfaces.
Erosive wear	Various particles, such as sand and slurries, impinge the surface.
Corrosive and oxidative wear	In corrosive wear, the fine corrosive products present on the surface make up the wear particles. When the corrosive layer is disrupted or eroded due to sliding or abrasion, a new layer begins to form, and this cycle of removal and corrosive layer formation repeats.

**Table 2:** A summary of SNC approaches used to modify the surfaces of ferrous alloys.

SNC Method	Description	Ref.
Surface Mechanical Attrition Treatment (SMAT)	It involves a chamber that vibrates within a frequency range of 50 Hz–20 kHz. Within this chamber, smooth ceramic or steel spherical shots, flying at velocities ranging from 1 to 20 m/s, bombard the surface of the alloy. This intense bombardment results in extremely high strain rates within the targeted alloy surface, ultimately leading to the formation of a nanostructured surface layer.	[3]
Shot Peening (SP)	This technology bears similarities to SMAT, with the key distinction being the size of the shot particles utilized in each process. In SP, the shot particles employed are of smaller diameters (0.2–1 mm) compared to SMAT (3–10 mm). Moreover, SP involves the high-velocity impact of these smaller shots onto the alloy surface, with the impact velocity being at least five times greater than that used in the SMAT technique.	[3]
Sandblasting (SB)	If micron-sized ceramic sands are employed instead of spherical balls in shot peening to achieve surface nanocrystallization of the alloy, the process is referred to as sandblasting (SB).	[3]
Nanopeening Treatment	Nanopeening treatment, also referred to as nanoscale surface peening, closely resembles the shot peening process with one notable distinction: the deliberate selection of operating conditions, including shot diameters, projected speed, and, most notably, the incidence angle. Unlike traditional shot peening, nanopeening utilizes an incidence angle that is no longer perpendicular to the alloy surface.	[19]
Supersonic Fine Particle Bombardment (SFPB)	This process entails the repetitive bombardment of the alloy surface with fine particles, each having a diameter of less than 200 μm, propelled by supersonically compressed air at velocities ranging from 300 to 1200 m/s.	[20]
Fast Multiple Rotation Rolling (FMRR)	It involves the rapid rotation and rolling of rotational tool tips on the metal surface while applying static pressure. Simultaneously, the alloy can move steadily back and forth in a horizontal direction.	[21]
High-Speed Pounding (HSP)	It involves the use of a pounding tool equipped with a high-speed tip that continually impacts the surface of the alloy	[22]
Ultrasonic Cold Forging Technology (UCFT)	A WC ball, attached to an ultrasonic horn device, impacts the alloy’s surface at a frequency of 20 kHz. The dynamic load applied during this process exhibits an amplitude 1.5–5 times greater than the constant static pressure. This method corresponds to Ultrasonic Impact Treatment (UIT) as documented in existing literature.	[23,24,25,26]
High-Frequency Impacting and Rolling (HFIR)	In this process, also known as the Ultrasonic Surface Rolling Process (USRP), a combination of constant force rolling, and ultrasonic impacting is utilized. The High-Frequency Impact Rolling (HFIR) tool converts oscillation into ultrasonic vibrations with the aid of a piezoelectric ceramic transducer. These ultrasonic vibrations are further amplified by an amplitude-changing rod. Concurrently, the HFIR tool advances along the processing path under a static force.	[27,28]
Surface mechanical rolling treatment (SMRT)	While a cylindrical sample rotates at a certain velocity, a polished cermet (WC/Co) ball is pressed into its surface to a specified penetration depth. Simultaneously, the ball slides along the sample axis from right to left at a specific velocity.	[29]

**Table 3:** Volume losses of samples during (corrosive) wear (×10^5^ µm^3^) [40].

Wear Test Type	304SS	Sandblasted 304SS	Sandblast-Annealed 304SS
Dry wear	6.90	3.39	2.70
Corrosive wear in NaCl solution	4.58	3.27	1.71

## Data Availability

No new data were created or analyzed in this study. Data sharing is not applicable to this article.
